# Improving the Ability of Image Sensors to Detect Faint Stars and Moving Objects Using Image Deconvolution Techniques

**DOI:** 10.3390/s100301743

**Published:** 2010-03-03

**Authors:** Octavi Fors, Jorge Núñez, Xavier Otazu, Albert Prades, Robert D. Cardinal

**Affiliations:** 1 Departament d’Astronomia i Meteorologia and Institut de Ciències del Cosmos (ICC), Universitat de Barcelona (UB/IEEC), Av. Diagonal 647, 08028 Barcelona, Spain; 2 Observatori Fabra, Barcelona, Spain; E-Mail: jorge@am.ub.es (J.N.); 3 Computer Vision Center, Computer Science Department, Universitat Autònoma de Barcelona, Campus UAB, Cerdanyola del Vallès, 08193 Barcelona, Spain; E-Mail: xotazu@cvc.uab.es (X.O.); 4 Departament d’Enginyeria del Terreny, Cartogràfica i Geofísica, Universitat Politècnica de Catalunya, Barcelona, Spain; E-Mail: albert@mercator.upc.edu (A.P.); 5 NEOSSat Mission, Department of Geoscience, University of Calgary, Calgary, Canada; E-Mail: rcardina@ucalgary.ca (R.D.C.)

**Keywords:** image processing, image deconvolution, faint stars, space debris, wavelet transform

## Abstract

In this paper we show how the techniques of image deconvolution can increase the ability of image sensors as, for example, CCD imagers, to detect faint stars or faint orbital objects (small satellites and space debris). In the case of faint stars, we show that this benefit is equivalent to double the quantum efficiency of the used image sensor or to increase the effective telescope aperture by more than 30% without decreasing the astrometric precision or introducing artificial bias. In the case of orbital objects, the deconvolution technique can double the signal-to-noise ratio of the image, which helps to discover and control dangerous objects as space debris or lost satellites. The benefits obtained using CCD detectors can be extrapolated to any kind of image sensors.

## 1. Introduction

In an image of the sky taken to observe either stars or orbital objects (satellites, space debris, *etc.*) the object of interest appears convolved with the structure and motion of the source, the emission process, the atmosphere, the telescope and detector interaction, *etc.* In a typical frame taken from the ground this convolution makes point-like stars appear as pixelized extended light spots, known as Point Spread Function (PSF), of the order of one arc second diameter. Such light distribution is in most cases well represented by a Gaussian-like pattern but it can vary along the image. If the object of interest is orbiting the Earth, the main effect is the convolution with its motion with respect to the stars. In this case, if the frame was taken tracking the sky, a point-like moving object appears in the images as a trail. The length of the trail depends on the relative speed of the object with respect to the Earth sidereal motion and on the exposure time. In both cases, the result of the convolution is to spread the light of the star or the moving object over several pixels of the image sensor decreasing the ability to be detected and the accuracy of the determination of its position.

Mathematically, the process can be described as an imaging equation that is a relationship between the light distribution of the source ***a*** (the ideal image) and ***p,*** the observed image by the sensor. After discretizing the problem the imaging equation can be written as: F*a + *b* + *n* = *p*, where ***a*** and ***p*** are the unknown and data vectors, respectively, and ***n*** and ***b*** are vectors representing the noise and the background in both object and data spaces, and ***F*** is a sparse matrix representing the PSF. In the case of the stars, the PSF can be understood as the actual image of a point-like star (mostly represented by a Gaussian-like pattern). In the case of orbital objects, the PSF is understood as the actual trailed image recorded on the frame by the moving satellite or space debris. Note that ***a***, ***p***, ***n*** and ***b*** represent, in general, two-dimensional images, but since they are discretized, they can be represented as vectors.

Image deconvolution consists of obtaining the best approximation of the ideal image ***a*** from the inversion of the imaging equation. However, it is well known this problem is an ill-posed noisy inverse one. Thus, the imaging equation cannot be correctly solved by linear methods such as matrix inversion or the direct Fourier inversion since these methods magnify the noise providing unacceptable results.

For a long time, image deconvolution was considered a luxury in optical astronomy, remote sensing or satellite tracking. However, since the discovery in 1990 of a severe problem of spherical aberration in the mirror of the Hubble Space Telescope (HST) a substantial amount of work has been done in this field directed towards optical and near-IR astronomy (with applications in other fields), covering different types of data noise and proposing dozens of algorithms.

## 2. Image Deconvolution Algorithms

The goal of image deconvolution is to extract from the sensor image ***p*** an approximation to the actual image ***a*** with reduced ripple, improved resolution (if possible) and making due allowance for noise. This involves some form of interpolation and extrapolation in the Fourier domain. A good algorithm should also give less weight to high-spatial-frequency data that can be corrupted by the noise and instead give more importance to extrapolated values obtained from better data at lower frequencies. In image deconvolution, the nature of the problem (including the kind of the noise) and the data itself (including the observation method) will lead to a different approach. Thus, there is no preferred algorithm to solve the image deconvolution problem and the different approaches are based on different instrumental noise models (Poisson, Gaussian, Poisson with Gaussian readout noise, *etc.*), on different statistical estimators (Maximum Likelihood, Bayesian, Maximum Entropy, *etc.*), on the nature of the object (point-like, extended or trailed) and even on the available computational resources.

Since in this paper it is not possible to review the dozens of developed algorithms, we direct the reader to the published excellent proceedings [1,2]; special issues [3] and reviews [4,5]. In these works the reader can find, among many others, algorithms based in Maximum Likelihood, Maximum Entropy and the Bayesian paradigm. Specific astrometric application to assess the recovery of faint stars was directed in [6–9]. A classical algorithm widely used in Astronomy is the Richardson-Lucy (R-L), with the two versions for Poisson data [10] and for Poisson data with Gaussian readout noise (as in many image sensors as the CCD cameras) [11,12]. However, we will focus our study into a more sophisticated algorithm—the Adaptive Wavelet-decomposition-based Maximum Likelihood (AWMLE) method [5,13], which shows a better noise amplification control and asymptotically convergence, and it is PSF-invariant like the majority of deconvolution algorithms that deal with large amount of data.

## 3. Detecting Faint Stars

Image deconvolution of astronomical frames makes substantial improvements in qualitative appearance of signal-to-noise ratio (SNR) and recovery of faint star images in terms of the number of detectable stars. As an example, we present the deconvolution of two different astronomical ground-based frames of the same telescope+sensor facility. In both examples we used the AWMLE algorithm and a hybrid PSF fitted from a collection of stars in the image. In particular, the sensor was a CCD camera with Poisson signal and Gaussian readout noise.

**Figure 1.**
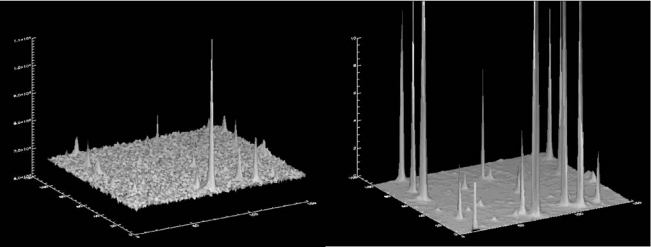
Plot showing a detail of a raw (left) and 140 AWMLE iterations deconvolved (right) image obtained with a retrofitted Baker-Nunn camera equipped with a CCD camera as image sensor. In order to ease the SNR comparison, the vertical axes were set to different ranges. In the case of the deconvolved image, up to six stars are out of range. The deconvolved image shows much larger SNR with respect to the raw image.

In the first example, a 149 × 103-pixel detail of the deconvolution of a large CCD image (4,096 × 4,096 pixels) obtained with the retrofitted Baker-Nunn camera of the Rothney Astrophysical Observatory in Canada (NESS-T BNC program) is shown. The Baker-Nunn cameras are modified Super-Schmidt Telescopes of 50cm aperture working at focal ratio f/1. To visually show the improvement attainable by deconvolution, Figure 1 shows a three dimensional plot of a detail of the raw image and its deconvolution after 140 iterations using the AWMLE algorithm and a hybrid (Moffat function core plus a corrective lookup table in the outer wings) PSF. The improvement in terms of resolution and SNR in the deconvolved image with respect to the raw data can be clearly seen.

**Figure 2.**
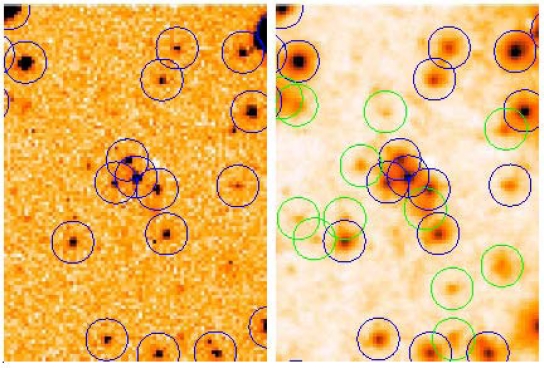
Raw image (left) and its AWMLE restoration (right) of an image obtained as in Figure 1 with the CCD camera of the NESS-T BNC program. Left: the detected stars are circled in blue. Right: the newly detected stars (not detected in the raw image) are circled in green. Both detection processes were performed with SExtractor algorithm [14], by using the same input parameters.

In the second example [8,9] a different detail from the same NESS-T BNC program is shown. Figure 2 shows the raw data and its deconvolution using the AWMLE algorithm at convergence (140 iterations) with the same hybrid type of the PSF used in Figure 1. A robust detection algorithm such as SExtractor [14] was used to detect stars in both frames. The same input parameters (detection threshold, FWHM of matching mask, *etc.*) were employed in both images for assuring fair comparison.

Note the remarkable increase in detected stars which was achieved: 12 newly detected stars were added to the 18 stars detected in the raw image. In the whole image (not shown in this paper) a total of 2,644 stars were detected and matched to a catalog in the restored image *versus* only 1,724 stars detected and matched in the original.

**Figure 3.**
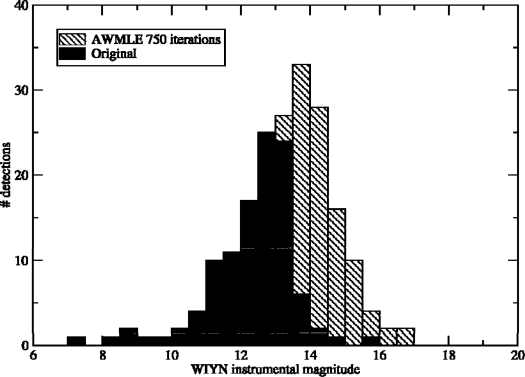
Magnitude histograms of matched detections for the original image and the AWMLE deconvolution at convergence of a QUEST image. Dashed area stands for the newly detected stars.

Alternatively, the gain in limiting magnitude can be estimated with the magnitude histograms of detected and matched (to a deeper catalog) detections for the original and deconvolved images. To show this, a new set of data has been considered. The data belong to an image obtained during the QUasar Equatorial Survey Team (QUEST) program using the 1.0-m Venezuelan Schmidt Telescope working in scanning (TDI) mode. Again a CCD camera was used as image sensor but, in this case, using the TDI observing technique. In Figure 3 [8], we overplot the magnitude histogram of the original image with the one from an AWMLE deconvolution once reached convergence. The dashed area in Figure 3 represents the stars detected only in the deconvolved image. In this particular data a total of 197 stars were detected (and matched to a catalog, extracted from a deeper image) *versus* only 109 stars detected and matched in the raw image. Considering the area of each histogram, which coincides with the number of matched detections, and using the simple expression Δ*m* = 2.5 log(N_deconvolved_/N_raw_) a limiting magnitude gain Δ*m* = 0.64 is derived.

Note that a gain of Δ*m* = 0.6 mag. means an increment of about 80% in available detections or, equivalently, a 32% in telescope aperture or, a decrease of 2.3-times in telescope cost without losing astrometric performance.

In summary, these results show that the benefit of using image deconvolution is equivalent to almost double the quantum efficiency of the used image sensor or to increase the effective telescope aperture by more than 30% (up to 40% in some cases).

With regard to the astrometric accuracy of the deconvolved images, the studies carried out in [6–9] showed that after deconvolution, the standard deviation of the Gaussian-fitted stellar intensity profiles (σ) are about half those obtained using the raw data. Since σ is directly related to the seeing present during the exposure, the results show that the deconvolved image can be considered equivalent to an image obtained with a seeing that is half of the original one i.e. under much better atmospheric conditions. The possibility of any systematic bias in the positions of the deconvolved stars with regard to the ones obtained using the raw images was ruled out using both synthetic data [7] and real data [8].

## 4. Detecting Faint Orbital Objects

Currently, there are millions of space debris orbiting the Earth. These include everything from entire spent rocket stages and defunct satellites to small lost satellites, explosion fragments, paint flakes, dust, and slag from solid rocket motors, coolant released by nuclear powered satellites, deliberate insertion of small needles and other small particles [15]. About 600,000 space debris objects are larger than 1 cm but only about 13,000 of them are cataloged. Space debris has become a growing concern in recent years, since collisions at orbital velocities can be highly damaging to functioning satellites and can also produce even more space debris. Even hypervelocity collisions between large satellites are likely. For example, on 10 February 2009 the Russian Cosmos 2251 slammed into the US Iridium 33, causing two large clouds of debris [15].

Image deconvolution can also play an important role to improve the ability of image sensors to detect and observe orbital objects as space debris. As stated above, if we take a frame tracking the sky, a moving object appears in the image as a trail. Hence, the light emitted by the orbital object is spread over a line of several pixels decreasing its SNR and, therefore, the chances to be detected and the accuracy in its position determination.

As in the case of stars, the idea behind the use of image deconvolution for increasing the detectability of trailing orbital objects in image sensors is to invert the imaging equation in order to concentrate the light spread along the trail in few pixels. To do this, we use the image of a trailing object as PSF.

**Figure 4.**
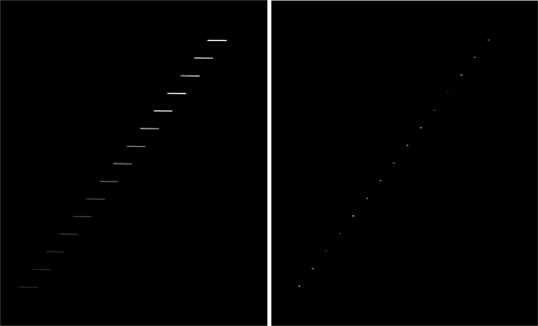
Simulated image of a collection of satellites (left) and its AWMLE deconvolution (right) using the trace of the brightest satellite as PSF. Both panels are displayed using the same grey scale distribution. The global flux of every trail is preserved in right panel while intensity distribution is compressed into much less pixels.

**Figure 5.**
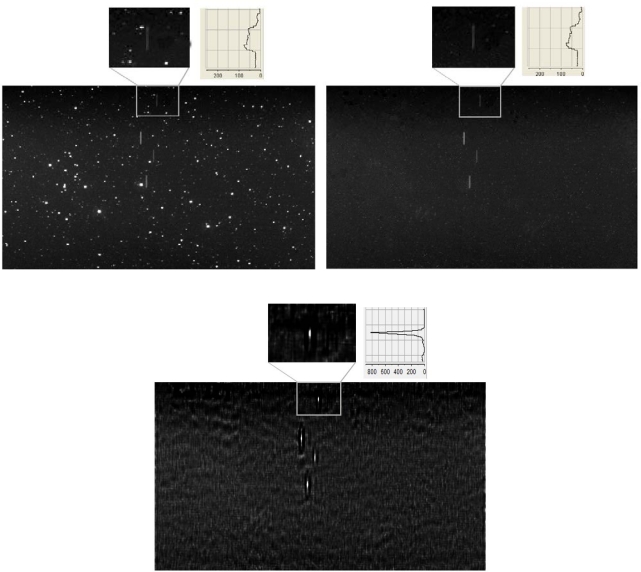
CCD image of a group of satellites (upper left). The same image with most of its stars removed (upper right). AWMLE deconvolution of upper right image using the brightest satellite trail as PSF (lower panel). All three panels are displayed using the same grey scale distribution. Note the high improvement in detectability of the faintest satellite (compare upper with lower zoomed panels). The light profiles show a large increase in the SNR of the deconvolved image with respect the original one.

To show the potentiality of the method, we performed the preliminary experiment depicted in Figure 4 and Figure 5.

In Figure 4, a simulated frame consisting of a collection of 15 trailing objects of decreasing intensity is generated. A deconvolution of such image using the AWMLE algorithm (200 iterations) and the brightest trail of left panel as PSF is performed. As clearly seen, the length of the original trailing objects is compressed in the central pixels, so that the SNR is improved. For example, in the faintest trailing object (lowest-left one), while in the original image the total flux is spread over about 90 pixels with an average intensity of 17 counts, in the deconvolved image this was concentrated in three pixels of about 520 counts each.

In Figure 5, we show a real image of a group of satellites taken by a retrofitted Baker-Nunn camera located in Hawaii [16] equipped with a CCD detector as image sensor. Point-like stars and the elongated trails left by a constellation of four satellites appear in the upper left panel of Figure 5. As in Figure 4, the idea is to use deconvolution using the trail left by the brightest satellite as PSF to increase the detectability of fainter satellites trails. However, deconvolution of upper left panel would lead to an image with stars showing distorted light distribution. Although such light profiles could be identified and eventually removed from the deconvolved image, it is preferable to proceed first by removing the stars from original image and second by performing the deconvolution of this intermediate image. In such a way, the detection of the satellites trails after deconvolution is much straightforward. In order to remove the stars from an image it is possible to use several techniques. One of them is the CLEAN algorithm [17], which is a very well-known and robust one in Astronomy. The CLEAN algorithm locates the profile of the star images and subtracts them in an iterative way. However, since the CLEAN algorithm could also mistakenly remove part of the trails left by the moving objects, another way to remove the stars from the image is to use a star catalog which gives the position on the image of the stars to be removed. These two steps are shown in upper right and lower panels of Figure 5. In order to remove the stars from upper left panel we made use of a combination of the two methods described above. As seen in upper right panel, the stars are mostly removed. In the second step, the AWMLE algorithm was employed to deconvolve, at convergence after 100 iterations, the upper right panel. As can be seen in the lower panel, the trail left by the faintest satellite (upper-left zoomed panel and upper-right zoomed panel) notably increases its SNR (lower zoomed panel), and therefore its detectability. Indeed, all the satellites trails are condensed into nearly point-like distributions, but we focused our attention only on the faintest one.

In order to numerically assess the result, we also show in the three zoomed panels the vertical scale in counts along the vertical direction. A quick inspection of the zoomed panels shows that in the upper panels the satellite trail peaks at 77 counts, while in the deconvolved lower panel the peak is around 800 counts. In terms of SNR, taking into account the background noise in both images, such increase represents a factor of no less than two, remarkably improving the chances to detect faint orbital space debris.

It is important to point out that, since the deconvolution concentrates the light of the trail in few pixels, the larger the trail the higher improvement in detectability would be obtained.

## 5. PSF Computation and Artifacts

The accuracy of the PSF to be used is a common limitation in most deconvolution situations. If large deviations exist between the chosen and actual PSFs, artifacts are likely to appear in the vicinity of brightest objects in deconvolved images [8]. However, the PSFs in this article were computed either by hybrid modeling (in the case of the stars) or by direct projection (in the case of the satellite trails). In both cases, they are likely to show very small deviations from the actual PSFs. Another possible approach to compute the PSF in the satellite trails case would be to consider a catalog of theoretical possible trails left in the image given the observing circumstances. Fortunately, the most important zones to control are the Geostationary ring (GO) and the Geostationary Transfer Orbits (GTO). To predict the motion of the orbital objects in these zones can be easily done. However, it is beyond the scope of this paper.

Finally, one of the main conclusions of [8] is that AWMLE asymptotically converges with a marginal number of false detections due to artifacts. Therefore, the number of artifacts introduced by the AWMLE algorithm by itself is negligible.

## 6. Conclusions

The techniques of image deconvolution can increase the performance of any combination of telescope and image sensor. In this paper we showed that image deconvolution can increase the ability to detect faint stars and faint space debris in CCD images. In the case of faint stars, an increment in the limiting magnitude of at least 0.6 magnitudes can be obtained without decreasing the astrometric accuracy or introducing any astrometric bias. It is interesting to note that such increment is equivalent to double the quantum efficiency of the image sensor (CCD detector), to increase the effective telescope aperture by more than a 30% or to decrease the telescope cost in a 2.3 factor. In the case of space debris and other orbital objects, the deconvolution technique also increases the signal-to-noise ratio of the image (in a factor of no less than two in the example of Figure 5), which can help to discover and control the growing population of dangerous objects as the space debris.
